# Insecticide resistance in disease vectors from Mayotte: an opportunity for integrated vector management

**DOI:** 10.1186/1756-3305-7-299

**Published:** 2014-07-01

**Authors:** Nicolas Pocquet, Frédéric Darriet, Betty Zumbo, Pascal Milesi, Julien Thiria, Vincent Bernard, Céline Toty, Pierrick Labbé, Fabrice Chandre

**Affiliations:** 1 Institut de Recherche pour le Développement, Unité Mixte de Recherche MIVEGEC (IRD 224-CNRS 5290-UM1-UM2), 911, avenue Agropolis, BP 64501 34394 Montpellier cedex 5, France; 2 Agence de Santé Océan Indien (ARS OI), St Denis, La Réunion Island, France; 3 Institut des Sciences de l’Evolution de Montpellier (UMR 5554, CNRS-UM2-IRD), Université Montpellier 2, Montpellier, France; 4 DASS Nouvelle Calédonie, Santé Environnementale, Nouméa, Nouvelle Calédonie

**Keywords:** Insecticide resistance, Mosquito control, Resistance management, Integrated vector management

## Abstract

**Background:**

Mayotte, a small island in the Indian Ocean, has been affected for many years by vector-borne diseases. Malaria, Bancroftian filariasis, dengue, chikungunya and Rift Valley fever have circulated or still circulate on the island. They are all transmitted by Culicidae mosquitoes. To limit the impact of these diseases on human health, vector control has been implemented for more than 60 years on Mayotte. In this study, we assessed the resistance levels of four major vector species (*Anopheles gambiae*, *Culex pipiens quinquefasciatus*, *Aedes aegypti* and *Aedes albopictus*) to two types of insecticides: i) the locally currently-used insecticides (organophosphates, pyrethroids) and ii) alternative molecules that are promising for vector control and come from different insecticide families (bacterial toxins or insect growth regulators). When some resistance was found to one of these insecticides, we characterized the mechanisms involved.

**Methods:**

Larval and adult bioassays were used to evaluate the level of resistance. When resistance was found, we tested for the presence of metabolic resistance through detoxifying enzyme activity assays, or for target-site mutations through molecular identification of known resistance alleles.

**Results:**

Resistance to currently-used insecticides varied greatly between the four vector species. While no resistance to any insecticides was found in the two *Aedes* species, bioassays confirmed multiple resistance in *Cx. p. quinquefasciatus* (temephos: ~ 20 fold and deltamethrin: only 10% mortality after 24 hours). In *An. gambiae*, resistance was scarce: only a moderate resistance to temephos was found (~5 fold). This resistance appears to be due only to carboxyl-esterase overexpression and not to target modification. Finally, and comfortingly, none of the four species showed resistance to any of the new insecticides.

**Conclusions:**

The low resistance observed in Mayotte’s main disease vectors is particularly interesting, because it leaves a range of tools useable by vector control services. Together with the relative isolation of the island (thus limited immigration of mosquitoes), it provides us with a unique place to implement an integrated vector management plan, including all the good practices learned from previous experiences.

## Background

Mayotte is a French island located in the Indian Ocean, in the Comoros archipelago. For many years, this island has been heavily affected by vector-borne diseases. Historically, the two diseases that mainly plagued the island were Bancroftian filariasis, mostly transmitted by *Culex pipiens quinquefasciatus*[1-4], and malaria, transmitted by several anopheline species, including *Anopheles gambiae s.s.*[5,6]. Today, malaria is still present in Mayotte, although the number of cases has significantly decreased during the last two years [7]. Moreover, while the disease was considered eliminated from the island, some cases of Bancroftian filariasis were recently recorded [8].

In addition to these endemic diseases, a major dengue fever outbreak in 1943 [9] and a chikungunya outbreak in 2005 and 2006 have also affected Mayotte [10]. Both are due to arboviruses transmitted by *Aedes* species. However, while dengue was principally transmitted by *Aedes aegypti*, chikungunya main vector was *Ae. albopictus*[11]. This last species, observed for the first time on the island in 2001 [12], has since almost completely replaced *Ae. aegypti*[13], and certainly played the main role in the recently recorded cases of dengue and chikungunya [14]. Finally, new arboviruses recently started to circulate on the island, including the Rift Valley Fever virus [15].

To limit the impact of these diseases on people from Mayotte, many vector control programs have been implemented since the early 50s [16]. Most of the efforts were intended to control *Cx. p. quinquefasciatus* and *An. gambiae* populations, to prevent filariasis and malaria. They relied almost entirely on the use of chemical insecticides (from the organochlorines (OC), organophosphates (OP) and pyrethroids (PYR) families), through extensive applications on larval breeding sites, indoor residual spraying treatments (IRS) [3,5,9,16-18] and, more recently, long-lasting insecticide treated nets (LLIN). These vector control campaigns have had good results and greatly limited the impact of lymphatic filariasis and malaria in Mayotte [4,6]. Today however, several constraints could impede vector control. The first constraint is administrative, with a significant reduction of the number of insecticides available for vector control due to new European^a^ regulations [19]. All pesticide molecules had indeed to be re-examined in 2007 for marketing authorization, through a costly application filed by the producers; some unprofitable yet efficient molecules were not supported. There are also technical difficulties, due to the increasing role of *Ae. albopictus* as a major vector of arboviruses in Mayotte. Due to their preferences for confined larval breeding sites (natural, like tree holes, or artificial, like used tires) and their eggs resistant to desiccation [20,21], *Ae. albopictus* is particularly difficult to reach through conventional sprays of insecticides. The third type of constraints is ecological: Mayotte is a small island with a specific ecosystem encompassing many endemic species, and as such must be protected from anthropic pollutions. The effects of insecticide treatments on non-target fauna and their potential accumulation in the food chain need to be taken into account and limited. Finally, the last and most important challenge come from evolutionary process: the long-term use of insecticides is known to select for resistance of the target insects, with the possible effect of rendering the available molecules ineffective for control [22].

However, in Mayotte, almost nothing was known on the resistance status of the various mosquito vectors, until a recent study on *Cx. p. quinquefasciatus*[23]. This study showed that many resistance mechanisms were present in this species, so that the lack of data for the other vectors became a major concern. In view of the history of insecticide treatments in the island, many resistance mechanisms could have been selected in the other species as well, and could prevent efficient vector control measures. There are indeed a large number of insecticide resistance mechanisms in mosquitoes, mainly through metabolic resistances or insecticide target modifications (review in: [24-26]). The usual way of overcoming resistance is to change the molecule used to restore efficient vector control. However, the number of new molecules available is continuously shrinking [27], and cross-resistance (i.e. the fact that one resistance mechanism is able to confer resistance to other molecule families) could lead to an additional reduction of alternatives [28].

All these constraints have to be considered to implement a rational and sustainable vector control plan. In this type of plan, it is clearly important to monitor the resistance levels to currently-used insecticides and to assay the few valuable and authorized molecules that could replace them in case of insecticide resistance development in the targeted vectors.

In this study, the four main mosquito vectors of the island (*Cx. p. quinquefasciatus*, *An. gambiae*, *Ae. aegypti* and *Ae. albopictus*) were thus investigated to determine their levels of resistance to the insecticides currently used in Mayotte: temephos (OP), *Bti* (bacterial toxins (BacT) extracted from *Bacillus thuringiensis* var *israelensis*), and deltamethrin (PYR). When resistance was found, the mechanisms involved were characterized through biochemical and molecular analyses. In addition, resistance to four candidate insecticides for vector control in Mayotte was also assayed: spinosad, an insecticide of bacterial origin (Spinosyns), and three insect growth regulators or IGRs, diflubenzuron, pyriproxyfen and methopren. The results are discussed in the light of the vector control strategies usable to prevent emergence and spread of resistance in the island vectors.

## Methods

### Mosquito samples and strains

Five laboratory strains were used in this study: *An. gambiae* KIS strain [29], *Cx. p. quinquefasciatus* SLAB strain [30], *Ae. aegypti* BORA strain [31] and *Ae. albopictus* PLP strain [32] were used as susceptible reference strains; the *An. gambiae* AcerKIS strain [33], homozygous for the G119S mutation of acetylcholinesterase [34], was used as the OP-resistant reference strain in this species.

**Figure 1:**
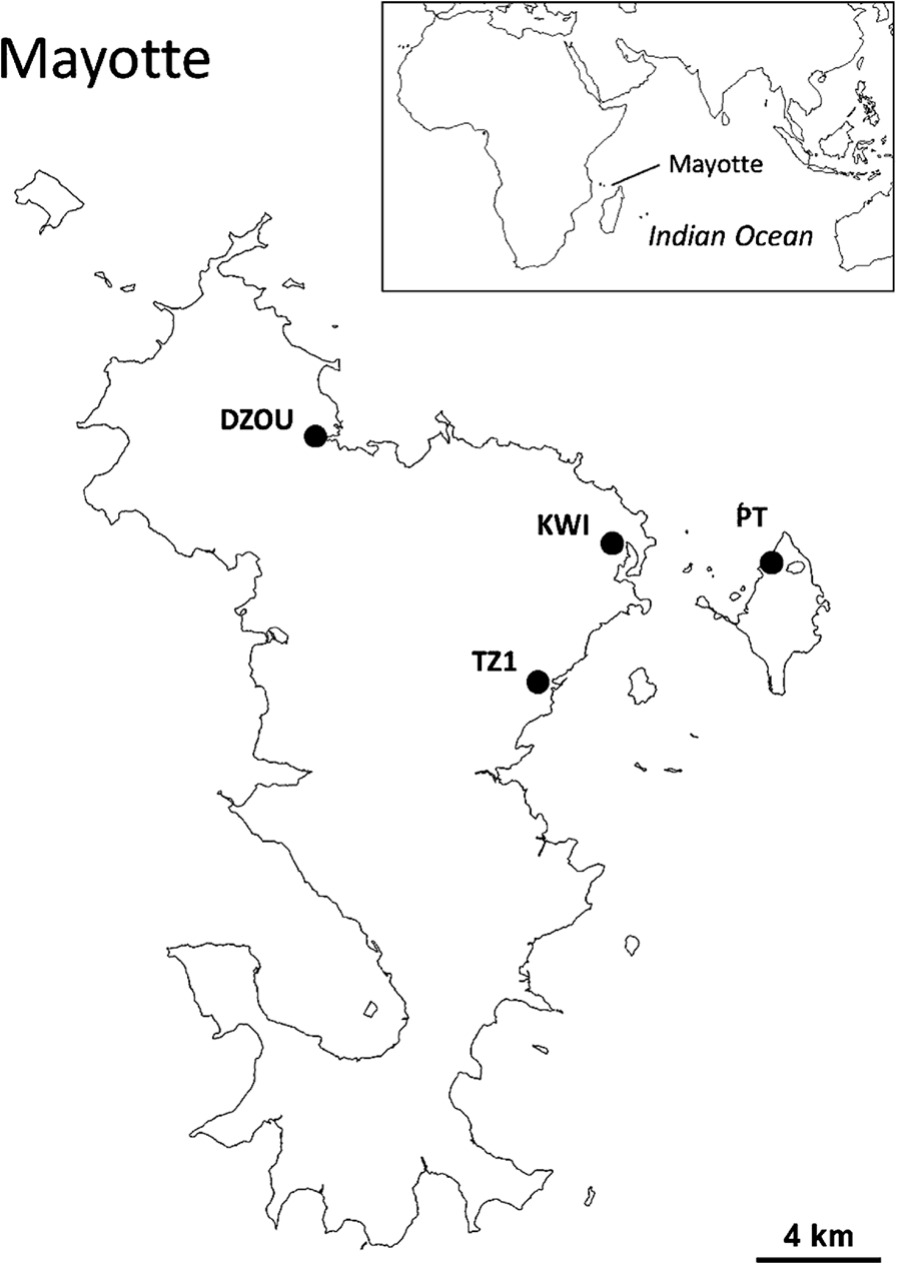
**Sampled populations in Mayotte.** Sampling was carried out in Dzoumogné for the DZOU colony of *An. gambiae*, in Tsoundzou 1 for TZ1 colony of *Cx. p. quinquefasciatus*, in Kaweni for KWI colony of *Ae. albopictus* and in Petite Terre for PT colony of *Ae. aegypti*.

Field larvae of *An. gambiae*, *Cx. p. quinquefasciatus*, *Ae. aegypti* and *Ae. albopictus* were collected in Mayotte between 2010 and 2011. Natural populations (Figure 1) were sampled from a garbage dump in Dzoumogné for *An. gambiae* (DZOU), an open sewer in Tsoundzou for *Cx. p. quinquefasciatus* (TZ1), several peri-domestic breeding sites in Petite Terre for *Ae. aegypti* (PT) and a stock of used tires in Kaweni for *Ae. albopictus* (KWI). The larvae of *An. gambiae* and *Cx. p. quinquefasciatus* were collected at early instars (1^st^ or 2^nd^), reared in the laboratory to 3^rd^ instar, and used for bioassays. The larvae of *Ae. aegypti* and *Ae. albopictus* were reared to adulthood in the laboratory. Mono-specific colonies of *Ae. aegypti* and *Ae. albopictus* were established, and females were blood-fed to obtain F1 offsprings, which were used for bioassays (3^rd^-instar larvae). In *Cx. p. quinquefasciatus*, *Ae. aegypti* and *Ae. albopictus* samples, some of the field larvae were kept, reared, and the adults were bred in the laboratory to establish TZ1, PT and KWI colonies. These colonies were used for IGR bioassays. Due to technical difficulties for establishing an *An. gambiae* colony from field individuals, IGR bioassays were directly performed on field-collected larvae.

Finally, to overcome the difficulties to establishing an *An. gambiae* colony while remaining close to the original field population (as for the other species), the DZOU temephos resistance gene(s) were introgressed into the genome of the KIS strain, leading to the DZKIS strain. DZOU males were crossed with unmated females of the KIS strain and their progeny reared in the laboratory. Third-instar larvae were selected with temephos at a dose killing 80% of the individuals. Male survivors were backcrossed on females of the KIS strain and selected again. The following generations were then left to cross among themselves, 3^rd^-instar larvae being selected with temephos at each generation, until the resistance level had stopped increasing (10 generations). This protocol provided the DZKIS strain, containing mainly DZOU genome (the field population colony), and just enough KIS genome to be lab-adapted. It also resulted in a strain more homogeneous in terms of resistance.

### Bioassays

Larval and adult bioassays were performed following WHO protocols [35,36]. Larval bioassays were carried out using ethanol solutions of the following active ingredients, temephos (OP), chlorpyrifos (OP), malathion (OP), propoxur (carbamate, or CM), spinosad (Spinosyns), diflubenzuron (IGR), pyriproxyfen (IGR) and methopren (IGR) (spinosad from Dow Agro Sciences, Indianapolis, USA; other products from Sigma-Aldrich, Germany), and using water solutions of *Bti* (BacT) formulation (Vectobac 12AS, 1200 ITU/mg). Larval bioassays were conducted on sets of 25 early 3^rd^-instar larvae placed in a cup with 99 ml of water. One ml of the tested insecticide solution was then added in each cup. Assays of four to nine doses in a minimum of two cups per dose were performed for each insecticide. Two replicates were performed for temephos, spinosad and *Bti*, and one or two replicates were performed for chlorpyrifos, malathion, propoxur and IGR insecticides (it results in 250 to 1500 mosquitoes assayed for each insecticide). In temephos, spinosad, chlorpyrifos, malathion, propoxur and *Bti* assays, larval mortality was recorded after 24 hours of insecticide exposure. For IGR assays, the total number of larvae in each cup was recorded after 24 hours and the number of emerging adults was recorded daily. Emergence Inhibition (EI) is calculated for each dose by subtracting the number of emerged adults to the total number of larvae at the beginning of the test. Note that in such IGR tests, regular feeding of larvae is required, due to their duration (over 10 days). For *Aedes* and *Culex* larvae, 3 to 5 mg per cup of a mixture of dog and fish foods were added every day. For *Anopheles* larvae, 0.5 to 1.5 mg per cup of fish food were added on the surface. The quantity of food was decreased at the appearance of the first pupae, as some larvae were still feeding.

Adult bioassays were carried out using WHO test tubes. This device allows exposing sets of 25 adult females (2–5 days old) to a filter paper impregnated with deltamethrin at a dose of 0.05% (products from Sigma-Aldrich, Germany). This diagnostic dose kills 100% of individuals in a susceptible population [37]. Four sets of 25 females were exposed for 60 minutes to deltamethrin to evaluate its knockdown effect (KD) on each colony or strain. Mortality was recorded after 24 hours. Two replicates per colony/strain were performed.

The analyses of dose-mortality responses were performed using the R software [38]. The R script BioRssay was used; it is freely available on the website of the Institut des Sciences de l’Evolution de Montpellier [39]. This script computes the doses of insecticide killing 50% and 95% of the tested colony or strain (Lethal Concentration 50 and 95, or LC_50_ and LC_95_) and the associated confidence intervals, using a script modified from Johnson *et al.*[40], which allows taking into account the heterogeneity of the data [41]. Mortality in controls is taken into account using the correction from the Abott’s formula [42]. A generalized linear model (GLM) with a binomial error and a probit link is then fitted to the data where the probit mortality is a function of the logarithm of the dose of insecticide for each colony/strain. The script also computes the slope and intercept of the regression for each colony/strain (and their standard errors), and tests for the linearity of the dose-mortality response (χ^2^ test). Finally, it allows the comparison of two or more strains or colonies and calculates the resistance ratios, i.e. RR_50_ or RR_95_ (=LC_50_ or LC_95_ of tested colony/LC_50_ or LC_95_ of the reference strain, resp.) and their 95% confidence intervals. A RR in which the confidence interval does not include 1 was considered as statistically significant, so that the tested colony was significantly more resistant than the reference. Note, however, that even slight differences between colonies/strains can be statistically significant, due to the high number of mosquitoes tested. However, even a statistically significant RR < 3 is usually considered of limited biological significance (such RR can be obtained when comparing susceptible strains, e.g. [43]), and we applied this criterion here. The script then builds custom graphs and a summary text file with the different parameters and tests is provided.

The same script was used to calculate the Emergence Inhibition Concentrations for IGR insecticides (EIC_50_ and EIC_95_) and the KnockDown Times for deltamethrin (KDT_50_ and KDT_95_).

### Metabolic resistance

Biochemical tests were performed on single 2–5 days-old females from the *An. gambiae* DZOU colony to evaluate the activity of the main families of detoxifying enzymes. Protein amount was quantified in microplates using the method of Bradford [44], the quantity or activity of the different detoxifying enzymes were expressed per mg of protein present in the homogenate or quantity of molecules metabolized per minute, respectively. Cytochrome P450 monooxygenases (named mixed function oxidases or MFO) were quantified indirectly by the peroxidase activity of the heme group with tetramethylbenzidine (note that all hemoproteins are thus quantified, not only MFO; [45]). Carboxyl-esterases (COE) were quantified indirectly by their ability to hydrolyze α-naphthyl and β-naphthyl acetate [46].

Statistical comparisons of detoxifying enzyme activities present in the *An. gambiae* susceptible strain KIS and the DZOU colony were computed using Mann–Whitney tests with the Statistica software [47].

### Analyses of target-site modifications

Total DNA of single mosquitoes of the *An. gambiae* DZOU colony was extracted using the CTAB protocol [48]. The G119S mutation, carried by the *ace-1*^*R*^ allele of the acetylcholinesterase-1 gene (AChE1), was investigated using the PCR-RFLP test described by Weill *et al*. [49]. Two substitutions in the *kdr* gene are known to cause resistance to PYR in *An. gambiae*: L1014F and L1014S, respectively most often encountered in West Africa and East Africa. They were investigated using the multiplex-PCR described in Martinez-Torres *et al.*[50] and Ranson *et al.*[51], respectively. We thereafter called these two alleles *kdr*^*R*^, indifferently. Only the L1014F mutation was found in *Culex quinquefasciatus* from Mayotte, where it was investigated in our precedent study [23], using the multiplex-PCR described in Martinez-Torres *et al.*[52]. The resistance allele was called *kdr*^*R*^ thereafter.

## Results and discussion

### No resistance observed in *Ae. aegypti* and *Ae. albopictus*

**Figure 2:**
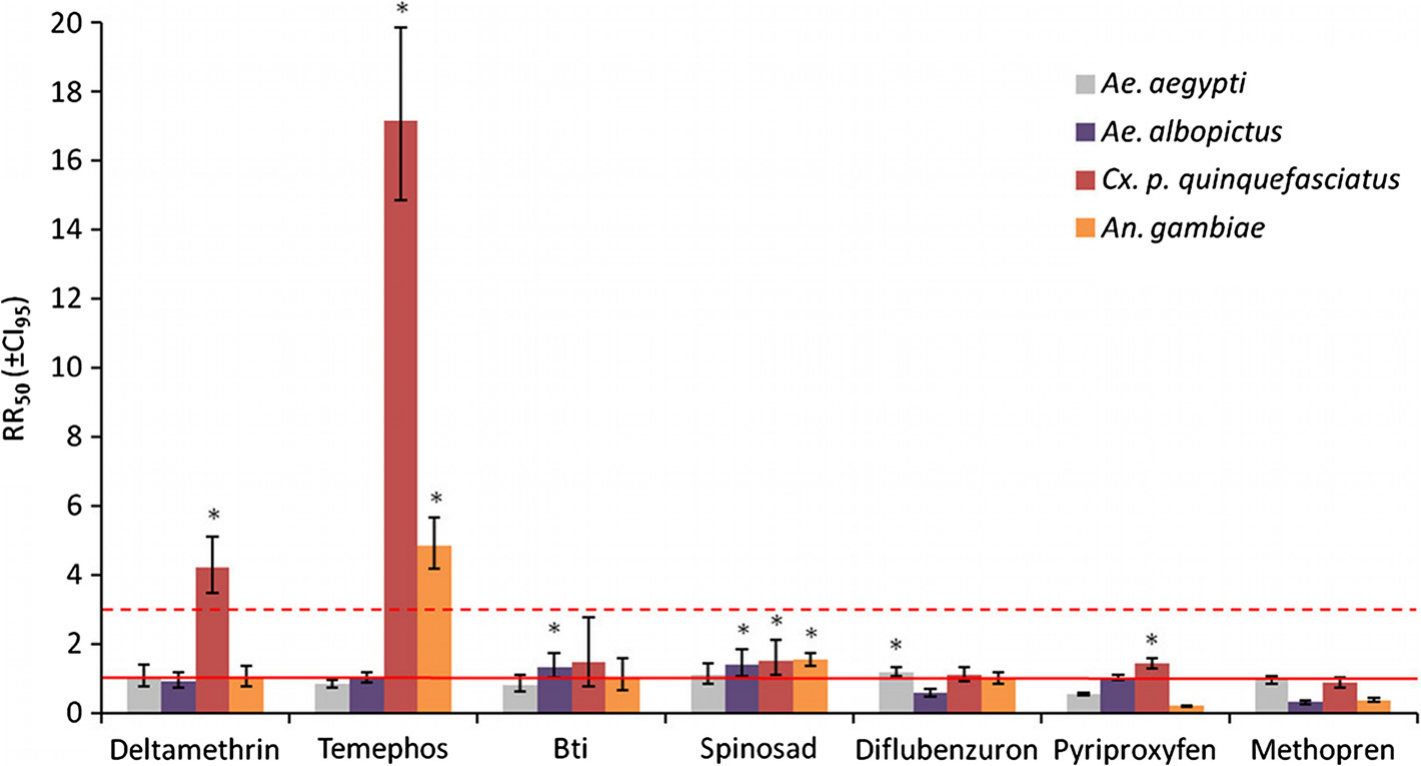
**Insecticide resistance in vector mosquitoes from Mayotte.** The resistance ratios (RR_50_, i.e. the ratios of LC_50_ of the tested colonies over the LC_50_ of the susceptible reference strain), of colonies from field populations of *Ae. aegypti* (gray), *Ae. albopictus* (purple) *Cx. p. quinquefasciatus* (red) and *An. gambiae* (orange) to different tested insecticides are presented. The error bars represent the confidence interval of RR at 95%. The solid red line represents RR = 1 (i.e. a LC_50_ equal to that of the susceptible reference) and the dotted red line represents RR = 3 (resistance is considered of biological significance when above). RR significantly higher than 1 (i.e. when CI_95_ does not include 1) are indicated by a star.

Larval bioassays revealed that colonies from field populations of *Ae. aegypti* and *Ae. albopictus* (PT and KWI, respectively) did not show biologically significant resistance to any of the tested larvicides (RR between 0.3 and 1.6, Figure 2 and Additional file 1). Similarly, adult bioassays to deltamethrin showed a complete susceptibility of these two species (over 97% mortality 24 hours after exposure) and no increase of knockdown times was observed as compared to the susceptible reference strains (RR between 0.9 and 1, Figure 2 and Additional file 2).

The susceptibility of these two *Aedes* species to IGRs, Spinosad and *Bti* is not surprising because these insecticides have never been used on the island before 2011 ([16]; Belon, personal communication). Since 2011, *Bti* has been used by the vector control service of Mayotte as a larvicide, but not against *Aedes* species.

In contrast, temephos (larvicide) was used in Mayotte from 1973 to 2012 and deltamethrin (adulticide) has been used since 1984 ([16], Belon, personal communication), but no resistance was observed in either *Ae. aegypti* or *Ae. albopictus*. Several factors may explain the absence of resistance to deltamethrin and temephos in these two species. First, before the 2005–2006 chikungunya outbreak [10], these species were not targeted by vector control treatments. Since the epidemic, control against these two vectors is essentially based on social mobilization and physical destruction of breeding sites. Only few insecticide treatments have therefore been carried out specifically against *Aedes* species in Mayotte. Secondly, their main breeding sites are peri-domestic containers used for water storage and small water collections in peri-urban areas (coconut, dead leaves, used tires, etc. [20,21]). These soil-less breeding sites are little affected by environmental xenobiotic contamination (insecticides or pollutants) and difficult to reach by the vector-control teams. Thirdly, deltamethrin is used in Mayotte either in Indoor Residual Spraying (IRS), or on Long Lasting Insecticide-treated Nets (LLIN). These two modes of treatment target adult female mosquitoes, but only indoors. *Ae. albopictus* and *Ae. aegypti* being diurnal and exophagous species [53,54], they are therefore not likely to be affected by LLINs, which protect people when sleeping. In addition, *Ae. albopictus* is an exophilic species [54], and although *Ae. aegypti* females can rest indoors, they do so preferentially on untreated surfaces [55,56], so that IRSs have little effect on these species. Overall, *Ae. albopictus* and *Ae. aegypti* are therefore likely to be subject to weak selection, which probably explains their complete susceptibility. This situation is radically different from that observed for some other French islands. For example, on the Martinique island, *Ae. aegypti* is the main target of vector control interventions, and this species presents strong levels of PYR resistance in this place [57]. A final remark concerning insensitive acetylcholinesterase target of OP and CM: it has been shown that, in these two *Aedes* species, the G119S mutation of this enzyme is highly unlikely, due to molecular constraints [58]. It was thus not surprising that this particular type of resistance was lacking, and it is unlikely to evolve in the future.

### High levels of resistance in *Cx. p. quinquefasciatus*

The results for *Cx. p. quinquefasciatus* are in sharp contrast to those of the two *Aedes* species. Larval and adult bioassays on TZ1 colony indeed revealed strong resistance respectively to temephos (RR_50_ = 17.2, RR_95_ = 18.9; Figure 2 and Additional file 1) and to deltamethrin (10% of mortality after 24 hours and a strong decrease of knockdown effect: RR_50_ = 4.2, RR_95_ = 4.9; Figure 2 and Additional file 2). However, no biologically significant resistance to any of the other tested insecticides has been identified in this colony (RR_50_ between 0.9 and 1.5, Figure 2 and Additional file 1), even if TZ1 colony showed a low resistance at LC_95_ to juvenile hormone analogs (pyriproxyfen and methopren, RR_95_ = 4.9 and 4.1 respectively).

The resistance mechanisms of *Cx. p. quinquefasciatus* in Mayotte have been studied in depth recently (see [23]). Two mechanisms of resistance to OPs were found on the island. The first was an overexpression of esterases, encoded by the *Ester*^*2*^ allele, and the second was a modification of the AChE1, due to the G119S mutation of the gene *ace-1*. Both were found at relatively high frequencies (0.59 for *Ester*^*2*^ and 0.39 for *ace-1*^*R*^; [23]). Similarly, the *kdr*^*R*^ allele, coding for a modification of the sodium channels allowing resistance to PYRs was found almost fixed on the entire island (*kdr*^R^ frequency = 0.98). Biochemical tests and bioassays with synergists did not reveal MFO involvement in PYR resistance [23]. The *kdr*^*R*^ allele thus appeared to be the main allele responsible for deltamethrin resistance, although the involvement of other metabolic resistance cannot be excluded. The low resistance to juvenile hormone analogues observed at the high doses could thus be due to the overproduction of esterases in this colony [23], as described in other insect species [59].

*Cx. p. quinquefasciatus* is the major vector of the Bancroftian filariasis, which has been plaguing Mayotte for many years [1,2,4]. Since the 50s, intense vector control efforts have been carried out against this species [16]. Many neurotoxic insecticides targeting AChE1 (OPs) and sodium channels (DDT followed by PYRs) have been used to control it [3,5,16-18]. These important selective pressures certainly explain the strong resistance to temephos and deltamethrin observed in the TZ1 colony. Such strong resistance to PYRs and OPs is not an isolated case in the Indian Ocean. Indeed, this species has been shown to also harbor major resistance mechanisms to PYR, OP and OC insecticides in Mauritius, Madagascar and La Réunion [23,32]).

The susceptibility of *Cx. p. quinquefasciatus* to *Bti* and spinosad and the low resistance to IGRs are probably related, similar to the *Aedes* species, in the fact that these insecticides have not been used in the past in Mayotte. They thus provide interesting alternatives to circumvent the high resistance to the insecticides classically used against *Culex*.

### An original temephos resistance mechanism in *An. gambiae*

*An. gambiae* has always been the main target of insecticide-based vector control in Mayotte, as malaria has been endemic on the island for many years [5,9,16,17,60]. In the DZOU colony, a significant resistance to temephos (RR_50_ = 4.8, RR_95_ = 12.9; Figure 2 and Additional file 1) was observed but there was no biologically significant resistance to any of the other insecticides (RR between 0.2 and 2).

Whereas the absence of resistance is expected for the larvicides that have never been used in the island before 2011 (*Bti*, spinosad, IGRs), absence of resistance to the adulticide deltamethrin is particularly striking (over 99% mortality after 24 hours, and full susceptibility to knockdown effect, RR between 1 and 1.1, Figure 2 and Additional file 2). Surprisingly, PCR performed on *An. gambiae* adult mosquitoes of the DZOU colony did not show either any known *kdr* resistance mutation, neither the western (L1014F substitution: N = 31, all susceptible homozygous) nor the eastern (L1014S substitution: N = 28, all susceptible homozygous). Insecticides that target the sodium channel have indeed been used in Mayotte since the early 70s and are still currently used. DDT (OC) was first used in 1973, to be replaced by deltamethrin (PYR) in the early 80s [16]. In several cases, the development of *An. gambiae s.s.* insecticide resistance has been associated with selection pressures related to the control of agricultural pests [29,61,62], but in Mayotte there are no areas of intense agriculture. One hypothesis to explain the lack of *kdr*^*R*^ alleles is thus that the selection pressure coming only from public health is not enough to maintain these alleles at a detectable level in natural populations. Furthermore, Mayotte is a relatively isolated island and a second hypothesis is that no importation of a *kdr*^*R*^ resistance allele has yet taken place. The fact that so far no *kdr*^*R*^ mutation has been reported in *An. gambiae* populations from the closest islands, especially in Madagascar [24,63], gives support to this second hypothesis.

**Figure 3:**
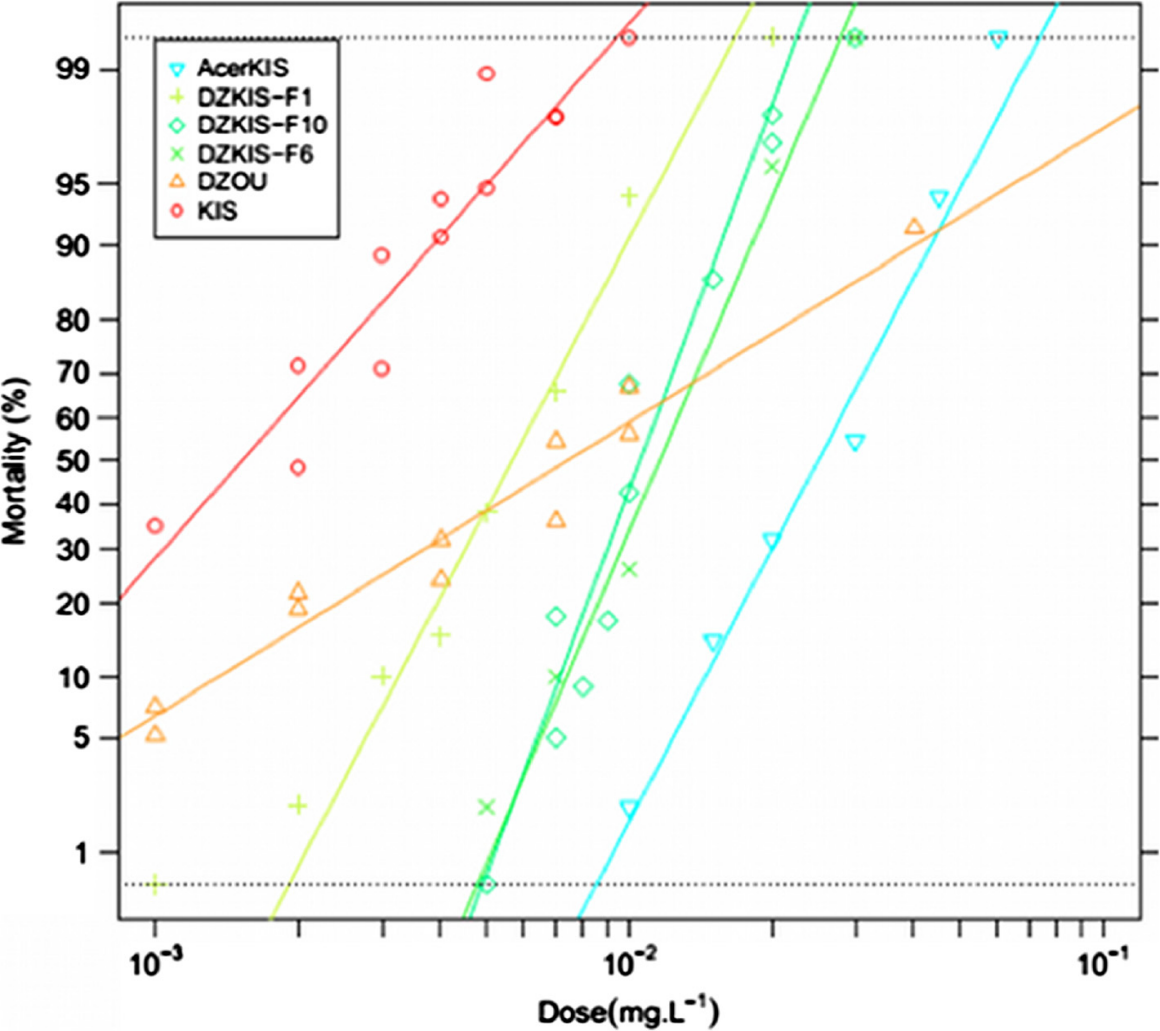
**Temephos resistance in the DZKIS strain.** The graph shows the evolution of the resistance level to temephos of the DZKIS strain in the 1^st^, 6^th^ and 10^th^ (i.e. the last) generations of selection. The dose-mortality of the DZOU original colony and of the KIS and AcerKIS reference strains (respectively susceptible and resistant to OPs through the G119S *ace-1* mutation) are also presented.

**Figure 4:**
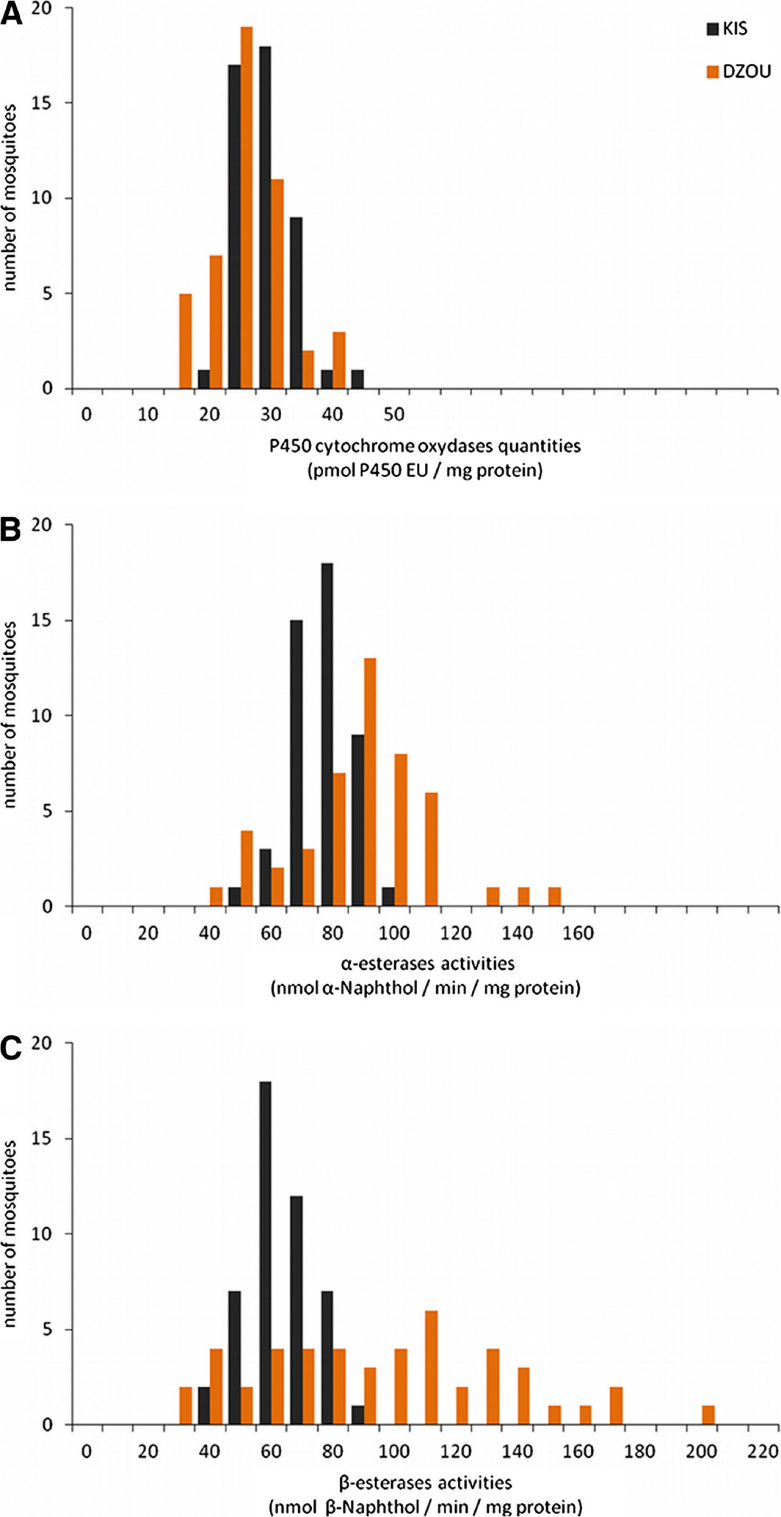
**Comparison of detoxification enzyme quantities or activities in single mosquitoes of KIS and DZOU.** The amount of cytochrome P450 oxidase **(A)** (MFO) is expressed in pmol of P450 Equivalent Unit per mg of protein for each mosquito. Activities of α **(B)** and β-esterases **(C)** (COE) are expressed as nmol of product formed (α or β-naphthol) per minute and per mg of protein.

As temephos has been used since 1973 in the island [16,17], the resistance to this insecticide observed in the DZOU colony is more expected. To better understand the mechanism(s) involved, the DZOU colony was partly introgressed in the reference susceptible strain KIS and selected at each generation with temephos, thereby creating the DZKIS strain, which carries a mainly DZOU genome but is able to be maintained in the laboratory. The results of this introgression are presented Figure 3. DZKIS temephos resistance was significantly higher than in DZOU sample at LC_50_ (RR_50_ = 6.9 and 4.8, respectively; Additional file 3), but lower at LC_95_ (RR_95_ = 3.5 versus 12.9, respectively; Additional file 3). This observation was mainly due to an increase of the slope of the dose-mortality regression between DZOU and DZKIS (1.75 and 7.58 respectively), reflecting a greater genetic homogeneity in the selected strain (due to selection at each generation). Tests carried out on DZKIS with other insecticides that target the AChE1 (Additional file 3) did not show biologically significant cross-resistance to chlorpyrifos (OP, RR_50_ = 1.2) and to malathion (OP, RR_50_ = 2.2). Moreover, the resistance levels of DZKIS were much lower than those of AcerKIS, the reference *ace-1*^*R*^ strain, for temephos (OP, RR_50_ = 6.9 vs 16.4, respectively), malathion (OP, RR_50_ = 2.2 vs 21.5, respectively) and propoxur (CM, RR_50_ = 5.6 vs ≈ 10 000). These results thus exclude the presence of insensitive AChE1 associated with G119S mutation. The absence of the *ace-1*^*R*^ allele was confirmed by PCR performed on adult mosquitoes from the DZOU original sample (N = 30, all homozygous for *ace-1* susceptible alleles). The activity or quantity of detoxifying enzymes in adult mosquitoes was compared between the DZOU sample and the KIS strain. The activities of α- and β-esterases were significantly higher in DZOU than in KIS (respectively, 1.19 and 1.47 fold, Mann–Whitney test: p < 0.001; Figure 4B and C). In contrast, the global quantity of MFO was significantly lower for DZOU than for KIS (0.90 fold, Mann–Whitney test: p < 0.001; Figure 4A).

In view of these results, it seems that the temephos resistance observed in *An. gambiae* from Mayotte is mainly due to COE overexpression or overactivity. Resistance to OPs and to a lesser extent CMs by COEs is commonly encountered in insects [64], particularly in mosquito vectors, such as *Culex* species [65,66] or *Ae. aegypti*[57,67,68]. This resistance mechanism usually confers a low level of resistance (about 10 fold, [26]), which is consistent with the resistance levels observed in DZOU and DZKIS (temephos and propoxur, 5 to 10 fold, Additional file 3). Although OP and CM resistance due to COE has already been reported in *An. gambiae*, it has so far always been found associated to the insensitive AChE1 [69,70], so that the situation in Mayotte is unique.

The DZOU colony breeding-site was a garbage dump, where a large variety of pollutants are present. This is a quite unexpected biotope for this species that usually prefers clean water collection. Such an environment, polluted by xenobiotics and organic matter, could have promoted the selection for an increase of COE expression, as it has been observed for other resistance mechanisms [71,72]. However, *An. gambiae* was also directly targeted by significant OP-based control in Mayotte [5,16]. The selective pressure generated by these treatments did not lead to the selection of the G119S *ace-1* mutation locally, and/or the allele was not imported, probably thanks to the island isolation (while it is extensively present in West Africa for example [33]). The contrast with *Cx. p. quinquefasciatus* is striking and will require more studies to be fully understood.

Finally, while temephos treatments have certainly favored overexpressed COE selection in the DZOU colony, they might also explain the lower MFO expression in this strain compared to KIS. Indeed, some OP, such as temephos, are bio-activated in their oxon form (the toxic form) by some oxidases, and it has been shown in *Cx. p. quinquefasciatus* that MFO were counter-selected in an environment under temephos pressure [73].

### Low resistance in disease vectors: an opportunity for Mayotte

In light of these results, the resistance status of vectors in Mayotte offers an unusual situation in the world of vector control. With the exception of temephos and deltamethrin resistances observed in *Cx. p. quinquefasciatus* and of the low temephos resistance in *An. gambiae*, the four main mosquito vector species were indeed susceptible to the majority of new tested insecticides (*Bti*, spinosad and two IGRs). Due to very different modes of action, resistance mechanisms to OPs and CM identified in *An. gambiae* and *Cx. p. quinquefasciatus*, including COEs, should not confer cross-resistance to these new insecticides (except, maybe, for juvenile hormone analogues, see above). Moreover, temephos has been recently abandoned from the arsenal of authorized insecticides for vector control in France due to European rules [19], and no other insecticides targeting AChE1 is presently authorized. As this resistance is costly in terms of fitness (e.g. [74,75]), they should thus disappear, and should not impact the future vector control efforts.

However, in order to preserve this positive situation, the usual vector control practices should be avoided. In particular, it is important to not use exclusively a single insecticide to control mosquitoes. *Bti* is currently the only larvicide used for vector control in Mayotte, thanks to its many advantages: this insecticide is highly specific, with little effect on non-target organisms [76], and it is a mixture of several synergistic toxins [77], thus limiting the risk of resistance development. Unfortunately, resistance has been described in a field population of *Cx. p. pipiens* from the United States [78] and resistance to separate *Bti* toxins in the laboratory were selected in *Ae. aegypti*[79] or *Cx. p. quinquefasciatus*[80]. Similarly, only deltamethrin is currently used for adulticides (IRS and LLINs). Its efficacy is preserved so far by the susceptibility of *An. gambiae*. However, this absence of resistance to PYRs should be carefully monitored, as it could rapidly spread through natural selection, following its appearance by mutation or importation [81]. Finally, even if other tested insecticides (spinosad and IGRs) are used less in vector control, examples of resistance to these compounds already exist in mosquitoes [78,82-84]. The exclusive use of any of those insecticides would therefore lead to the rapid emergence and selection of resistance in mosquitoes from Mayotte.

To prevent the development of resistance in these disease vectors, various resistance management strategies can be used. One of the most efficient strategies is to alternatively use insecticides with different modes of action and for which no cross-resistance occur in target populations [26,27]. Such strategies require a large enough panel of molecules. This may be a problem since in Mayotte, as mentioned before, *Bti* is the only larvicide currently allowed for use in natural breeding sites with non-target fauna associated, and deltamethrin the only adulticide authorized. Moreover, alternatives would be necessary in case of emergence of resistance. Therefore, a change in the national, but also European, policies regarding pesticides agreement would be much welcome. Some molecules could be re-authorized to be used only in case of public-health threat for example. This may be the case for temephos, which is a handy, low-cost and relatively safe molecule [85]. Although low resistance to this insecticide was observed in Mayotte (*Anopheles* and *Culex*), the operational doses could remain mostly effective against these vectors [86]. This molecule could thus be used as a back-up in case of emergency. Again, it is important to stress that such back-up would not mean using a single molecule in less pressing periods, in which case emergencies would become the rule.

More generally, the absence of strong resistance in most vectors allows the local vector control programme to develop a preemptive and reasoned insecticide use strategy in order to prevent the risk of development of resistance. This is very positive as such strategies are most often only considered in dire circumstances, i.e. when resistance is installed and when they are thus the least effective. However, the fight against mosquito disease vectors in Mayotte should not be exclusively based on insecticides, but should rather follow an Integrated Vector Management strategy (IVM [87]). This strategy recommends the combination of several tools to manage vector populations: physical destruction of breeding sites, social mobilization of communities, entomological monitoring and rational use of insecticides by all those implementing any action [26,87]. A recent study compared 61 vector control interventions against dengue vectors and showed that interventions based on IVM were more effective than interventions based only on environmental management, biological control or chemical control alone [88]. IVM has already shown good results against *Ae. aegypti* in Singapore and Vietnam [89,90]. This strategy requires the collaboration of several health sectors (vector control services, epidemiologists, hospitals), but also of other sectors not directly related to health (local administration, urbanization development, immigration surveys, waste management, etc.). For example, most of the breeding sites of *Cx. p. quinquefasciatus* in Mayotte are open sewers and latrines. Improving wastewater management and personal sanitation could greatly reduce the number of available breeding sites for this species. Similarly, the forthcoming closing of the garbage dump of Dzoumogné would limit the number of breeding sites for *An. gambiae* in this area.

## Conclusion

Mayotte is an ideal territory to implement an IVM approach and to carefully anticipate vector control management. Indeed, the economic development of the island is now fast and many public works are ongoing. It would be relatively easy to integrate the concept of vector management in the land and city planning policies. Moreover, social mobilization is already used by the local vector control services and is continuously improved. Finally, the low levels of insecticide resistance observed in the main mosquito vectors of the island allow usage of most of the larvicide and adulticide tested here. Thus, only anticipated resistance management strategies and regular entomological surveys remain to be implemented. This unusual situation allows being relatively optimistic about the future of vector control in Mayotte.

## Endnote

^a^NB: Mayotte has recently become a French overseas administrative department and has to comply with Biocide Directive 98/8/EC.

## Abbreviations

OC: Organochlorines; OP: Organophosphates; CM: Carbamat; PYR: Pyrethroids; BacT: Bacterial toxins; *Bti*: *Bacillus thuringiensis* var *israelensis*; IGR: Insect growth regulator; LC: Lethal concentration; EI: Emergence inhibition; EIC: Emergence inhibition concentration; KD: Knockdown; KDT: Knockdown times; RR: Resistance ratio; GLM: Generalized linear model; MFO: Mixed function oxidases; COE: Carboxyl-esterases; AChE1: Acetylcholinesterase-1; IRS: Indoor residual spraying; LLIN: Long lasting insecticide-treated net; IVM: Integrated vector management.

## Competing interests

The authors declare that they have no competing interest.

## Authors’ contributions

Conceived and designed the experiments: NP, FD, BZ, FC. Performed the experiments: NP, FD, VB, CT. Analyzed the data: NP, PM, PL, FC. Contributed to reagents, materials and analysis tools: BZ, JT, FC. Wrote the manuscript: NP, PL, FC. All authors read and approved the final version of the manuscript.

## Supplementary Material

Additional file 1 **Effects of larvicides on mosquito vectors from Mayotte.** Resistance levels of DZOU, TZ1, PT and KWI colonies to temephos, *Bti*, spinosad, diflubenzuron, pyriproxyfen and methopren are compared to resistance levels of the reference strains KIS and AcerKIS, SLAB, BORA and PLP, respectively. For *An. gambiae*, additional tests with chlorpyrifos, malathion and propoxur are presented. N is the total number of tested larvae. The 50 and 95% lethal concentrations (LC_50_ and LC_95_) and the 50 and 95% emergence inhibition concentrations (EIC_50_ and EIC_95_) are expressed in mg/l, with their associated confidence intervals at 95% (CI_95_). Finally, the corresponding resistant ratios (RR), i.e. the ratios of LC or EIC of the tested colony over the susceptible reference strain, are also indicated and presented in bold when significantly higher than 1 (i.e. when CI_95_ does not include 1). Click here for file

Additional file 2 **Effect of deltamethrin on adult vector mosquitoes from Mayotte.** Short-term knockdown effect and mortality at 24 hours induced by deltamethrin on DZOU, TZ1, PT, KWI colonies and KIS, SLAB, BORA and PLP reference strains are presented. N is the total number of tested adult females. The 50 and 95% knockdown times (KDT_50_ and KDT_95_) are expressed in minutes, with their associated confidence intervals at 95% (CI_95_). Finally, the corresponding resistant ratios (RR), i.e. the ratios of KDT of the tested colony over the susceptible reference strain, are also indicated and presented in bold when significantly higher than 1 (i.e. when CI_95_ does not include 1). Click here for file

Additional file 3 **Effects of OP and CM larvicides on *Anopheles gambiae* from Mayotte.** Resistance levels of the introgressed DZKIS strain are compared to resistance levels of the reference strains KIS and AcerKIS for three OP (temephos^a^, chlorpyrifos, malathion) and one CM (propoxur) larvicides. N is the total number of tested larvae. The 50 and 95% lethal concentrations (LC_50_ and LC_95_) are expressed in mg/l, with their associated confidence intervals at 95% (CI_95_). Finally, the corresponding resistant ratios (RR), i.e. the ratios of LC of the tested colony over the susceptible reference strain, are also indicated and presented in bold when significantly higher than 1 (i.e. when CI_95_ does not include 1). Click here for file
