# Supplementary material for: Insecticide resistance in disease vectors from Mayotte: an opportunity for integrated vector management
Source: Parasit Vectors. 2014 Jul 1;7:299. doi: 10.1186/1756-3305-7-299 (PMC4094441; doi:10.1186/1756-3305-7-299)
Supplement: Additional file 1 — Effects of larvicides on mosquito vectors from Mayotte. Resistance levels of DZOU, TZ1, PT and KWI colonies to temephos, Bti, spinosad, diflubenzuron, pyriproxyfen and methopren are compared to resistance levels of the reference strains KIS and AcerKIS, SLAB, BORA and PLP, respectively. For An. gambiae, additional tests with chlorpyrifos, malathion and propoxur are presented. N is the total number of tested larvae. The 50 and 95% lethal concentrations (LC50 and LC95) and the 50 and 95% emergence inhibition concentrations (EIC50 and EIC95) are expressed in mg/l, with their associated confidence intervals at 95% (CI95). Finally, the corresponding resistant ratios (RR), i.e. the ratios of LC or EIC of the tested colony over the susceptible reference strain, are also indicated and presented in bold when significantly higher than 1 (i.e. when CI95 does not include 1). [file 1756-3305-7-299-S1.pdf]

AdditionalFile1: Effects of larvicides on mosquito vectors from Mayotte.

**Species**	**Insecticide**	**Strain**	**N**	**LC$_{50}$ / EIC$_{50}$ (CI$_{95}$)**	**LC$_{95}$ / EIC$_{95}$ (CI$_{95}$)**	**Slope**	**RR$_{50}$ (CI$_{95}$)**	**RR$_{95}$ (CI$_{95}$)**
An. gambiae	Temephos	KIS	1164	1.5x10^{-3} (1.2x10^{-3} – 1.8x10^{-3})	5.0x10^{-3} (4.1x10^{-3} – 6.7x10^{-3})	3.17	-	-
		DZOU	1080	7.4x10^{-3} (6.1x10^{-3} – 9.1x10^{-3})	6.4x10^{-2} (4.1x10^{-2} – 1.3x10^{-1})	1.75	**4.84 (4.16 - 5.64)**	**12.9 (7.68 - 21.6)**
	Bti	KIS	900	1.9x10^{-1} (1.4x10^{-1} – 2.4x10^{-1})	6.1x10^{-1} (4.1x10^{-1} – 1.87)	3.23	-	-
		DZOU	1000	1.9x10^{-1} (1.7x10^{-1} – 2.1x10^{-1})	5.1x10^{-1} (4.3x10^{-1} – 6.7x10^{-1})	3.9	1.02 (0.64 - 1.6)	0.83 (0.05 - 13.7)
	Spinosad	KIS	1289	3.2x10^{-3} (2.7x10^{-3} – 3.8x10^{-3})	1.7x10^{-2} (1.3x10^{-2} – 2.5x10^{-2})	2.27	-	-
		DZOU	1367	5.0x10^{-3} (3.7x10^{-3} – 6.5x10^{-3})	3.1x10^{-2} (2.0x10^{-2} – 6.1x10^{-2})	2.07	**1.54 (1.37 - 1.73)**	**1.8 (1.31 - 2.48)**
	Diflubenzuron	KIS	496	1.7x10^{-3} (1.5x10^{-3} – 2.0x10^{-3})	3.5x10^{-3} (2.9x10^{-3} – 4.9x10^{-3})	5.34	-	-
		DZOU	416	1.7x10^{-3} (6.9x10^{-4} – 2.7x10^{-3})	5.6x10^{-3} (3.2x10^{-3} – 3.81)	3.19	0.99 (0.84 - 1.16)	1.59 (0.99 - 2.55)
	Pyriproxyfen	KIS	595	5.1x10^{-5} (3.3x10^{-5} – 7.3x10^{-5})	5.5x10^{-4} (3.4x10^{-4} – 1.2x10^{-3})	1.6	-	-
		DZOU	507	9.9x10^{-6} (1.7x10^{-6} – 1.7x10^{-5})	1.2x10^{-4} (6.1x10^{-5} – 1.0x10^{-3})	1.54	0.19 (0.17 - 0.22)	0.21 (0.17 - 0.26)
	Methopren	KIS	285	1.9x10^{-3} (1.2x10^{-3} – 3.0x10^{-3})	1.5x10^{-2} (7.5x10^{-3} – 5.7x10^{-2})	1.84	-	-
		DZOU	594	7.1x10^{-4} (3.6x10^{-4} – 1.2x10^{-3})	2.9x10^{-2} (1.1x10^{-2} – 1.8x10^{-1})	1.02	0.38 (0.32 - 0.45)	**1.95 (1.04 - 3.63)**
Cx. p. quinquefasciatus	Temephos	SLAB	994	1.5x10^{-3} (1.3x10^{-3} – 1.7x10^{-3})	3.3x10^{-3} (2.7x10^{-3} – 4.8x10^{-3})	4.59	-	-
		TZI	1174	2.5x10^{-2} (2.2x10^{-2} – 2.8x10^{-2})	6.3x10^{-2} (5.2x10^{-2} – 8.4x10^{-2})	4.1	**17.2 (14.9 - 19.9)**	**18.9 (12.8 - 28.1)**
	Bti	SLAB	986	1.8x10^{-1} (6.1x10^{-2} – 2.5x10^{-1})	6.6x10^{-1} (4.7x10^{-1} – 2.28)	2.94	-	-
		TZI	1302	2.7x10^{-1} (2.4x10^{-1} – 3.0x10^{-1})	6.9x10^{-1} (5.9x10^{-1} – 8.7x10^{-1})	3.97	1.46 (0.77 - 2.77)	1.04 (0.04 - 28)
	Spinosad	SLAB	994	6.8x10^{-2} (5.9x10^{-2} – 7.9x10^{-2})	2.7x10^{-1} (2.1x10^{-1} – 3.6x10^{-1})	2.77	-	-
		TZI	1184	1.0x10^{-1} (8.8x10^{-2} – 1.2x10^{-1})	4.5x10^{-1} (3.4x10^{-1} – 6.6x10^{-1})	2.57	**1.52 (1.11 - 2.09)**	1.69 (0.37 - 7.77)
	Diflubenzuron	SLAB	244	2.2x10^{-3} (9.9x10^{-4} – 3.4x10^{-3})	6.6x10^{-3} (3.9x10^{-3} – 4.7x10^{-1})	3.45	-	-
		TZI	1326	2.4x10^{-3} (1.2x10^{-3} – 3.3x10^{-3})	1.2x10^{-2} (7.5x10^{-3} – 3.9x10^{-2})	2.44	1.11 (0.93 - 1.33)	**1.75 (1.05 - 2.91)**
	Pyriproxyfen	SLAB	697	1.9x10^{-5} (1.6x10^{-5} – 2.2x10^{-5})	6.3x10^{-5} (4.9x10^{-5} – 8.9x10^{-5})	3.18	-	-
		TZI	1495	2.7x10^{-5} (2.0x10^{-5} – 3.5x10^{-5})	3.1x10^{-4} (2.3x10^{-4} – 4.4x10^{-4})	1.57	**1.43 (1.3 - 1.57)**	**4.88 (4.14 - 5.74)**
	Methopren	SLAB	248	7.1x10^{-4} (4.0x10^{-4} – 1.2x10^{-3})	5.6x10^{-3} (2.8x10^{-3} – 2.8x10^{-2})	1.83	-	-
		TZI	895	6.2x10^{-4} (3.8x10^{-4} – 8.8x10^{-4})	2.3x10^{-2} (1.4x10^{-2} – 4.8x10^{-2})	1.05	0.87 (0.73 - 1.04)	**4.06 (2.48 - 6.64)**
Ae. aegypti	Temephos	BORA	998	4.2x10^{-3} (4.1x10^{-3} – 4.3x10^{-3})	6.1x10^{-3} (5.9x10^{-3} – 6.4x10^{-3})	10	-	-
		PT	978	3.5x10^{-3} (2.9x10^{-3} – 4.2x10^{-3})	6.4x10^{-3} (5.1x10^{-3} – 1.2x10^{-2})	6.46	0.84 (0.74 - 0.96)	1.04 (0.78 - 1.38)
	Bti	BORA	1200	1.0x10^{-1} (9.3x10^{-2} – 1.1x10^{-1})	2.7x10^{-1} (2.3x10^{-1} – 3.3x10^{-1})	3.91	-	-
		PT	1001	8.1x10^{-2} (7.2x10^{-2} – 9.0x10^{-2})	2.1x10^{-1} (1.8x10^{-1} – 2.7x10^{-1})	4.04	0.81 (0.6 - 1.08)	0.78 (0.29 - 2.11)

	Spinosad	BORA	1187	5.5x10^{-2} (5.0x10^{-2} – 6.0x10^{-2})	1.2x10^{-1} (1.1x10^{-1} – 1.6x10^{-1})	4.61	-	-
		PT	787	6.0x10^{-2} (4.7x10^{-2} – 7.6x10^{-2})	1.2x10^{-1} (8.7x10^{-2} – 3.3x10^{-1})	5.58	1.09 (0.83 - 1.42)	0.94 (0.44 - 2.01)
	Diflubenzuron	BORA	1187	1.3x10^{-3} (7.3x10^{-4} – 1.9x10^{-3})	5.8x10^{-3} (3.2x10^{-3} – 8.2x10^{-2})	2.55	-	-
		PT	995	1.6x10^{-3} (1.2x10^{-3} – 1.9x10^{-3})	3.3x10^{-3} (2.6x10^{-3} – 5.9x10^{-3})	5.08	**1.17 (1.05 - 1.31)**	0.56 (0.42 - 0.74)
	Pyriproxyfen	BORA	594	6.8x10^{-5} (5.5x10^{-5} – 9.2x10^{-5})	1.8x10^{-4} (1.2x10^{-4} – 4.8x10^{-4})	3.84	-	-
		PT	1290	3.6x10^{-5} (2.8x10^{-5} – 5.1x10^{-5})	2.9x10^{-4} (1.6x10^{-4} – 9.4x10^{-4})	1.83	0.54 (0.49 - 0.59)	**1.6 (1.23 - 2.07)**
	Methopren	BORA	999	1.1x10^{-3} (7.2x10^{-4} – 1.5x10^{-3})	4.1x10^{-3} (2.4x10^{-3} – 1.5x10^{-2})	2.81	-	-
		PT	1195	1.0x10^{-3} (8.7x10^{-4} – 1.1x10^{-3})	3.5x10^{-3} (2.8x10^{-3} – 5.0x10^{-3})	2.99	0.94 (0.85 - 1.05)	0.87 (0.65 - 1.16)
Ae. albopictus	Temephos	PLP	1084	6.2x10^{-3} (5.8x10^{-3} – 6.7x10^{-3})	9.6x10^{-3} (8.4x10^{-3} – 1.2x10^{-2})	8.82	-	-
		KWI	1205	6.3x10^{-3} (6.0x10^{-3} – 6.6x10^{-3})	9.7x10^{-3} (8.8x10^{-3} – 1.1x10^{-2})	8.62	1.01 (0.88 - 1.16)	1.02 (0.71 - 1.47)
	Bti	PLP	1185	6.2x10^{-2} (5.6x10^{-2} – 6.7x10^{-2})	1.7x10^{-1} (1.5x10^{-1} – 2.0x10^{-1})	3.76	-	-
		KWI	1199	8.2x10^{-2} (7.2x10^{-2} – 9.2x10^{-2})	2.0x10^{-1} (1.7x10^{-1} – 2.5x10^{-1})	4.37	**1.33 (1.02 - 1.74)**	1.16 (0.58 - 2.31)
	Spinosad	PLP	1200	6.6x10^{-2} (5.9x10^{-2} – 7.2x10^{-2})	1.6x10^{-1} (1.4x10^{-1} – 1.9x10^{-1})	4.38	-	-
		KWI	992	9.2x10^{-2} (8.2x10^{-2} – 1.0x10^{-1})	2.5x10^{-1} (2.1x10^{-1} – 3.5x10^{-1})	3.73	**1.4 (1.06 - 1.84)**	1.63 (0.64 - 4.12)
	Diflubenzuron	PLP	372	2.6x10^{-3} (2.0x10^{-3} – 1.0x10^{-2})	5.9x10^{-3} (3.6x10^{-3} – 2.73)	4.62	-	-
		KWI	1000	1.5x10^{-3} (1.3x10^{-3} – 1.7x10^{-3})	3.2x10^{-3} (2.7x10^{-3} – 4.2x10^{-3})	5	0.57 (0.48 - 0.68)	0.53 (0.31 - 0.93)
	Pyriproxyfen	PLP	943	6.4x10^{-5} (5.0x10^{-5} – 9.0x10^{-5})	4.9x10^{-4} (2.6x10^{-4} – 1.7x10^{-3})	1.87	-	-
		KWI	1000	6.6x10^{-5} (5.3x10^{-5} – 8.4x10^{-5})	4.1x10^{-4} (2.4x10^{-4} – 1.1x10^{-3})	2.08	1.02 (0.95 - 1.1)	0.83 (0.63 - 1.1)
	Methopren	PLP	498	1.3x10^{-3} (7.8x10^{-4} – 2.6x10^{-3})	1.0x10^{-2} (4.0x10^{-3} – 1.14)	1.83	-	-
		KWI	498	4.0x10^{-4} (2.8x10^{-4} – 5.0x10^{-4})	2.9x10^{-3} (2.2x10^{-3} – 4.7x10^{-3})	1.89	0.3 (0.25 - 0.36)	0.28 (0.17 - 0.49)

Resistance levels of colonies from field populations (DZOU, TZ1, PT and KWI) to temephos, *Bti*, spinosad, diflubenzuron, pyriproxyfen and methopren are compared to resistance levels of the reference strains KIS and AcerKIS, SLAB, BORA and PLP, respectively. N is the total number of tested larvae. The 50 and 95% lethal concentrations (LC_{50} and LC_{95}) and the 50 and 95% emergence inhibition concentrations (EIC_{50} and EIC_{95}) are expressed in mg/l, with their associated confidence intervals at 95% (CI_{95}). Finally, the corresponding resistant ratios (RR), i.e. the ratios of LC or EIC of the tested colony over the susceptible reference strain, are also indicated and bolded when significantly higher than 1 (i.e. when CI_{95} does not include 1).
