# Supplementary material for: Insecticide resistance in disease vectors from Mayotte: an opportunity for integrated vector management
Source: Parasit Vectors. 2014 Jul 1;7:299. doi: 10.1186/1756-3305-7-299 (PMC4094441; doi:10.1186/1756-3305-7-299)
Supplement: Additional file 2 — Effect of deltamethrin on adult vector mosquitoes from Mayotte. Short-term knockdown effect and mortality at 24 hours induced by deltamethrin on DZOU, TZ1, PT, KWI colonies and KIS, SLAB, BORA and PLP reference strains are presented. N is the total number of tested adult females. The 50 and 95% knockdown times (KDT50 and KDT95) are expressed in minutes, with their associated confidence intervals at 95% (CI95). Finally, the corresponding resistant ratios (RR), i.e. the ratios of KDT of the tested colony over the susceptible reference strain, are also indicated and presented in bold when significantly higher than 1 (i.e. when CI95 does not include 1). [file 1756-3305-7-299-S2.pdf]

Additional File 2: Effect of deltamethrin on adult vector mosquitoes from Mayotte.

Species	Strain/ colony	N	KDT_{50} (CI_{95})	KDT_{95} (CI_{95})	Slope	RR_{50} (CI_{95})	RR_{95} (CI_{95})	Mortality at 24H
An. gambiae	KIS	211	11 (10 - 11)	16 (15 - 17)	10.3	-	-	100%
	DZOU	191	11 (10 - 12)	17 (16 - 19)	8.19	1.01 (0.76 - 1.35)	1.11 (0.73 - 1.69)	99%
Cx. p. quinquefasciatus	SLAB	201	13 (13 - 14)	20 (19 - 21)	9.13	-	-	94%
	TZI	183	56 (54 - 57)	98 (92 - 106)	6.73	**4.21 (3.46 - 5.11)**	**4.88 (3.47 - 6.85)**	10%
Ae. aegypti	BORA	198	11 (11 - 11)	16 (15 - 16)	11	-	-	100%
	PT	201	11 (11 - 12)	16 (15 - 17)	11.59	1.03 (0.76 - 1.39)	1.01 (0.66 - 1.55)	100%
Ae. albopictus	PLP	195	14 (14 - 15)	22 (21 - 23)	8.93	-	-	95%
	KWI	202	13 (13 - 13)	19 (18 - 20)	10.62	0.92 (0.73 - 1.17)	0.86 (0.62 - 1.2)	97%

Short-term knockdown effect and mortality at 24 hours induced by deltamethrin on DZOU, TZ1, PT and KWI colonies and KIS, SLAB, BORA and PLP reference strains are presented. N is the total number of tested adult females. The 50 and 95% knockdown times (KDT_{50} and KDT_{95}) are expressed in minutes, with their associated confidence intervals at 95% (CI_{95}). Finally, the corresponding resistant ratios (RR), i.e. the ratios of KDT of the tested colony over the KDT of the susceptible reference strain, are also indicated and bolded when significantly higher than 1 (i.e. when CI_{95} does not include 1).
