# Supplementary material for: Insecticide resistance in disease vectors from Mayotte: an opportunity for integrated vector management
Source: Parasit Vectors. 2014 Jul 1;7:299. doi: 10.1186/1756-3305-7-299 (PMC4094441; doi:10.1186/1756-3305-7-299)
Supplement: Additional file 3 — Effects of OP and CM larvicides on Anopheles gambiae from Mayotte. Resistance levels of the introgressed DZKIS strain are compared to resistance levels of the reference strains KIS and AcerKIS for three OP (temephosa, chlorpyrifos, malathion) and one CM (propoxur) larvicides. N is the total number of tested larvae. The 50 and 95% lethal concentrations (LC50 and LC95) are expressed in mg/l, with their associated confidence intervals at 95% (CI95). Finally, the corresponding resistant ratios (RR), i.e. the ratios of LC of the tested colony over the susceptible reference strain, are also indicated and presented in bold when significantly higher than 1 (i.e. when CI95 does not include 1). [file 1756-3305-7-299-S3.pdf]

Additional File 3: Effects of OP and CM larvicides on *Anopheles gambiae* from Mayotte.

Insecticide	Strain	N	LC_{50} (CI_{95})	LC_{95} (CI_{95})	Slope	RR_{50} (CI_{95})	RR_{95} (CI_{95})
Temephos	KIS	1164	$1.5x10^{-3}$ ($1.2x10^{-3}$ – $1.8x10^{-3}$)	$5.0x10^{-3}$ ($4.1x10^{-3}$ – $6.7x10^{-3}$)	3.17	-	-
	DZOU	1080	$7.4x10^{-3}$ ($6.1x10^{-3}$ – $9.1x10^{-3}$)	$6.4x10^{-2}$ ($4.1x10^{-2}$ – $1.3x10^{-1}$)	1.75	**4.84 (4.16 - 5.64)**	**12.9 (7.68 - 21.6)**
	DZKIS	1106	$1.1x10^{-2}$ ($9.6x10^{-3}$ – $1.2x10^{-2}$)	$1.7x10^{-2}$ ($1.5x10^{-2}$ – $2.3x10^{-2}$)	7.58	**6.93 (5.84 - 8.21)**	**3.46 (2.47 - 4.85)**
	AcerKIS	302	$2.5x10^{-2}$ ($2.2x10^{-2}$ – $2.9x10^{-2}$)	$5.1x10^{-2}$ ($4.1x10^{-2}$ – $7.3x10^{-2}$)	5.38	**16.4 (12.2 - 22.2)**	**10.1 (5.29 - 19.2)**
Chlorpyrifos	KIS	1199	$7.2x10^{-4}$ ($6.7x10^{-4}$ – $7.7x10^{-4}$)	$1.4x10^{-3}$ ($1.2x10^{-3}$ – $1.5x10^{-3}$)	6.06	-	-
	DZKIS	1041	$8.7x10^{-4}$ ($7.7x10^{-4}$ – $9.7x10^{-4}$)	$1.7x10^{-3}$ ($1.4x10^{-3}$ – $2.1x10^{-3}$)	5.83	**1.21 (1.08 - 1.35)**	**1.24 (1.02 - 1.5)**
Malathion	KIS	840	$1.9x10^{-2}$ ($1.8x10^{-2}$ – $2.2x10^{-2}$)	$4.4x10^{-2}$ ($3.7x10^{-2}$ – $6.0x10^{-2}$)	4.56	-	-
	DZKIS	492	$4.3x10^{-2}$ ($3.6x10^{-2}$ – $5.3x10^{-2}$)	$6.8x10^{-2}$ ($5.5x10^{-2}$ – $1.4x10^{-1}$)	8.29	**2.23 (1.69 - 2.95)**	1.52 (0.74 - 3.11)
	AcerKIS	348	$4.1x10^{-1}$ ($2.6x10^{-1}$ – $6.4x10^{-1}$)	1.51 (0.90 – 4.95)	2.93	**21.5 (6.15 - 75.2)**	33.9 (0.3 - 3766)
Propoxur	KIS	588	$1.5x10^{-2}$ ($1.1x10^{-2}$ – $1.8x10^{-2}$)	$4.9x10^{-2}$ ($3.8x10^{-2}$ – $7.1x10^{-2}$)	3.15	-	-
	DZKIS	841	$8.1x10^{-2}$ ($7.6x10^{-2}$ – $8.8x10^{-2}$)	$1.2x10^{-1}$ ($1.1x10^{-1}$ – $1.5x10^{-1}$)	9.62	**5.57 (4.07 - 7.62)**	**2.48 (1.23 - 4.99)**
	AcerKIS	494	144 (129 – 159)	243 (209 – 317)	7.26	**9872 (7511 - 12975)**	**4988 (3138 - 7927)**

Resistance levels of the introgressed DZKIS strain are compared to resistance levels of the reference strains KIS and AcerKIS for three OP (temephos[a], chlorpyrifos, malathion) and one CM (propoxur) larvicides. N is the total number of tested larvae. The 50 and 95% lethal concentrations (LC_{50} and LC_{95}) are expressed in mg/l, with their associated confidence intervals at 95% (CI_{95}). Finally, the corresponding resistant ratios (RR), i.e. the ratios of LC of the tested strain over the LC_{50} of the susceptible reference strain, are also indicated and bolded when significantly higher than 1 (i.e. when CI_{95} does not include 1).

[a]DZOU results for temephos have been reported here from Additional File 1 for easier comparison.
